# Prevalence of Obesity Among Youths by Household Income and Education Level of Head of Household — United States 2011–2014

**DOI:** 10.15585/mmwr.mm6706a3

**Published:** 2018-02-16

**Authors:** Cynthia L. Ogden, Margaret D. Carroll, Tala H. Fakhouri, Craig M. Hales, Cheryl D. Fryar, Xianfen Li, David S. Freedman

**Affiliations:** ^1^Division of Health and Nutrition Examination Surveys, National Center for Health Statistics, CDC; ^2^Office of Analysis and Epidemiology, National Center for Health Statistics, CDC; ^3^Division of Nutrition, Physical Activity and Obesity, National Center for Chronic Disease Prevention and Health Promotion, CDC.

Obesity prevalence varies by income and education level, although patterns might differ among adults and youths (*1*–*3*). Previous analyses of national data showed that the prevalence of childhood obesity by income and education of household head varied across race/Hispanic origin groups (*4*). CDC analyzed 2011–2014 data from the National Health and Nutrition Examination Survey (NHANES) to obtain estimates of childhood obesity prevalence by household income (≤130%, >130% to ≤350%, and >350% of the federal poverty level [FPL]) and head of household education level (high school graduate or less, some college, and college graduate). During 2011–2014 the prevalence of obesity among U.S. youths (persons aged 2–19 years) was 17.0%, and was lower in the highest income group (10.9%) than in the other groups (19.9% and 18.9%) and also lower in the highest education group (9.6%) than in the other groups (18.3% and 21.6%). Continued progress is needed to reduce disparities, a goal of *Healthy People 2020*. The overall *Healthy People 2020* target for childhood obesity prevalence is <14.5% (*5*).

NHANES is a cross-sectional survey designed to monitor the health and nutritional status of the civilian noninstitutionalized U.S. population (*6*). The survey consists of in-home interviews and standardized physical examinations conducted in mobile examination centers. The NHANES sample is selected using a complex, multistage probability design. During 2011–2014, non-Hispanic black, non-Hispanic Asian, and Hispanic persons, among other groups, were oversampled. Any non-Hispanic person reporting more than one race was included in an “other” category and included in the total estimates but not reported separately. The NHANES response rate for youths aged <20 years was 77.6% during 2011–2012 and 76.1% during 2013–2014. During the physical examination, standardized measurements of weight and height were obtained. Body mass index (BMI) was calculated as weight in kilograms divided by height in meters squared, rounded to the nearest 10th. Obesity among youths was defined as a BMI at or above the age- and sex-specific 95th percentile of the 2000 CDC growth charts (https://www.cdc.gov/growthcharts/cdc_charts.htm).

Household income was defined using FPL information, which accounts for inflation and family size (https://aspe.hhs.gov/prior-hhs-poverty-guidelines-and-federal-register-references) and categorized as ≤130%, >130% to ≤350%, and >350% of FPL. The cut-off point for participation in the Supplemental Nutrition Assistance Program is 130% of FPL, and 350% provides relatively equal sample sizes for each income group. Education was defined using education level of head of household and was categorized as a high school graduate or less, some college, and college graduate.

All estimates accounted for the complex survey design including examination sample weights. Confidence intervals for estimates were constructed using the Korn and Graubard method (*7*). Differences between groups were tested using a 2-sided univariate t statistic (p<0.05). Linear and quadratic trends from 1999–2002 to 2011–2014 were conducted using 4-year survey cycles. Pregnant females and persons with missing weight or height were excluded (139) for a total sample size of 6,878 during 2011–2014. For estimates by FPL another 517 persons were missing data and were excluded from analyses of FPL; for estimates by education level, 224 persons were missing data and were excluded from analyses of education.

Overall, 17.0% of youths aged 2–19 years had obesity during 2011–2014 (Table). The prevalence was 18.9% among those in the lowest income group, 19.9% among those in the middle group, and 10.9% among those in the highest income group. Among females, patterns in non-Hispanic white, non-Hispanic Asian, and Hispanic youths were similar, with the prevalence of obesity lower in the highest income group than in both other groups, but the differences by income were statistically significant only among non-Hispanic white females. Obesity prevalence did not differ by income among non-Hispanic black females. Among males, there was a lower obesity prevalence in the highest income group only in non-Hispanic Asian youths (compared with the lowest income group) and Hispanic youths (compared with both other income groups).

**TABLE:** Prevalence of obesity among youths (persons aged 2–19 years), by race/Hispanic origin, sex, household income, and education of household head — National Health and Nutrition Examination Survey, United States, 2011–2014

Characteristic	No.	% (95% CI)				
		All	Race/Hispanic origin			
			White, non-Hispanic	Black, non-Hispanic	Asian, non-Hispanic	Hispanic
**Total**	**6,878**	**17.0 (15.5–18.6)**	**14.7 (12.3–17.3)**	**19.5 (17.1–22.2)**	**8.6 (6.4–11.2)**	**21.9 (20.0–23.9)**
Females	3,371	17.1 (15.1–19.3)	15.1 (11.7–19.1)	20.7 (17.1–24.6)	5.3 (2.9–8.6)	21.4 (18.8–24.1)
Males	3,507	16.9 (15.1–19.0)	14.3 (11.2–17.9)	18.4 (16.1–21.0)	11.8 (8.3–16.1)	22.4 (19.9–24.9)
**Household income relative to federal poverty level**						
**Total**						
≤130%	3,131	18.9 (17.3–20.6)	15.5 (12.8–18.5)	19.4 (17.0–22.0)	13.2 (8.2–19.7)	22.8 (19.4–26.5)
>130% to ≤350%	1,974	19.9 (16.8–23.3)	18.0 (12.6–24.6)	19.9 (15.5–25.0)	8.9 (4.9–14.6)	23.7 (19.4–28.5)
>350%	1,256	10.9 (8.0–14.4)*^,†^	11.0 (7.3–15.7)	19.8 (12.2–29.4)	4.4 (1.9–8.4)*^,§^	11.8 (7.5–17.4)*^,†^
**Females**						
≤130%	1,539	19.7 (17.4–22.1)	17.8 (13.3–23.1)	19.9 (15.7–24.6)	8.4 (2.6–19.1)^¶^	22.5 (18.9–26.3)
>130% to ≤350%	969	21.5 (16.9–26.8)	21.2 (13.0–31.6)	21.6 (16.3–27.6)	8.2 (2.4–19.0)^¶^	22.7 (17.0–29.2)
>350%	613	8.0 (5.0–12.0)*^,†^	7.2 (3.5–12.8)*^,†^	21.1 (9.6–37.2)	1.3 (0.1–4.8)^¶^	13.8 (6.3–25.2)
**Males**						
≤130%	1,592	18.1 (15.5–21.0)	13.5 (9.2–18.7)	19.0 (15.7–22.6)	18.0 (10.1–28.6)	23.1 (18.0–28.9)
>130% to ≤350%	1,005	18.4 (15.6–21.4)	15.0 (10.0–21.2)	18.1 (12.1–25.5)	9.5 (3.9–18.7)^§^	24.6 (20.0–29.7)
>350%	643	13.7 (9.5–18.8)	14.7 (9.2–21.9)	18.7 (12.1–26.9)	7.6 (2.8–16.0)*^,§^	10.0 (4.8–17.9)*^,†^
**Education level of head of household **						
**Total**						
High school graduate or less	3,254	21.6 (20.0–23.3)	19.6 (16.2–23.3)	21.1 (17.5–25.0)	13.2 (8.5–19.3)	24.2 (20.9–27.7)
Some college	1,936	18.3 (15.4–21.5)**	17.6 (12.4–23.9)	19.7 (16.3–23.4)	12.0 (6.0–20.7)	19.9 (16.2–23.9)
College graduate	1,464	9.6 (7.3–12.5)**^,††^	8.5 (5.8–12.1)**^,††^	15.4 (9.8–22.5)	5.5 (3.1–8.9)**	13.5 (6.9–22.8)**
**Females**						
High school graduate or less	1,583	22.7 (20.7–24.9)	22.5 (17.5–28.1)	21.0 (16.0–26.7)	9.2 (4.4–16.5)	23.9 (20.1–28.0)
Some college	938	18.3 (14.6–22.6)**	18.0 (11.8–25.7)	22.1 (17.4–27.4)	8.0 (1.3–23.7)^¶^	17.3 (12.5–23.0)**
College graduate	739	8.5 (5.5–12.4)**^,††^	7.5 (3.9–12.8)**^,††^	16.3 (10.2–24.1)	3.3 (0.7–9.2)^¶^	14.0 (6.8–24.3)**
**Males**						
High school graduate or less	1,671	20.6 (18.1–23.2)	16.9 (11.6–23.3)	21.1 (17.5–25.1)	16.9 (9.0–27.7)	24.4 (20.5–28.7)
Some college	998	18.3 (14.7–22.4)	17.3 (11.0–25.3)	17.2 (13.4–21.6)	14.6 (6.7–26.4)	22.3 (15.9–29.8)
College graduate	725	10.7 (7.6–14.7)**^,††^	9.6 (5.5–15.2)**	14.5 (6.9–25.4)	7.9 (3.8–14.0)	12.9 (5.8–23.9) ^§,^**

**Abbreviation:** CI = confidence interval.

* Significantly different from ≤130% of FPL, p<0.05.

^†^ Significantly different from >130% to ≤350% of FPL, p<0.05.

^§^ Estimate might be unreliable because relative standard error is between 30% and 40%.

^¶^ Estimate might be unreliable because relative standard error is >40%.

** Significantly different from high school graduate or less, p<0.05.

^††^ Significantly different from some college, p<0.05.

Among youths, the prevalence of obesity decreased with increasing level of education of the head of household: 21.6% (high school graduate or less), 18.3% (some college), and 9.6% (college graduate). The same pattern was seen overall and in females and males in all race-Hispanic origin groups, but differences were not significant for non-Hispanic black youths (total, male, or female) or non-Hispanic Asian males or females.

From 1999–2002 to 2011–2014 the prevalence of obesity increased among females in the two lowest income groups (Figure 1). There was a nonsignificant decrease in obesity prevalence among females in the highest income group, and the difference in childhood obesity prevalence between the lowest and highest income groups increased over time. Among males, a quadratic trend was observed in the lowest income group: obesity prevalence was 16.9% during 1999–2002, increased to 21.0% during 2007–2010, and then declined to 18.1% during 2011–2014. The difference in prevalence between the lowest and highest income groups did not change over time for males.

**FIGURE 1:**
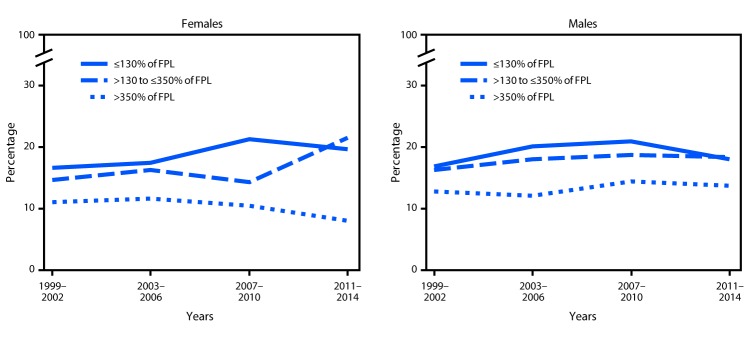
Trends*^,†^ in obesity prevalence among youths (persons aged 2–19 years), by household income — National Health and Nutrition Examination Survey, United States, 1999–2002 through 2011–2014 **Abbreviation:** FPL = federal poverty level. * Linear trend (p<0.05) for females ≤130% of FPL, >130% to ≤350% of FPL. ^†^ Quadratic trend (p<0.05) for males ≤130% of FPL.

Obesity prevalence among youths increased from 1999–2002 to 2011–2014 among females and males in households headed by persons with the least education (high school graduate or less) and among females in households headed by persons with some college education. There were no other significant trends. In addition, the difference in childhood obesity prevalence between the lowest and highest head of household education groups increased over time for females but not for males (Figure 2).

**FIGURE 2:**
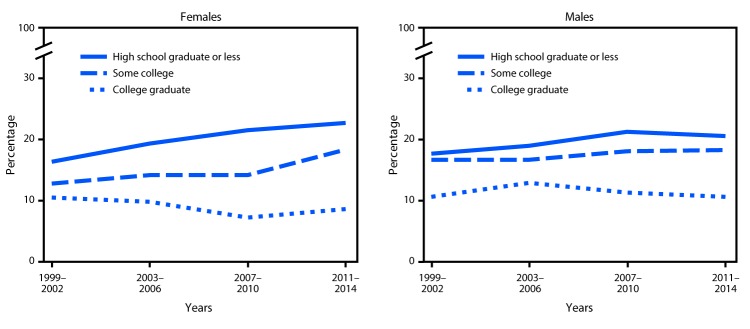
Trends* in prevalence of obesity among youths (persons aged 2–19 years), by education level of head of household — National Health and Nutrition Examination Survey, United States, 1999–2002 through 2011–2014 * Linear trend (p<0.05) for females, high school graduate or less and some college, and males, high school graduate or less.

## Discussion

During 2011–2014, the relationships between childhood obesity and income and childhood obesity and education of household head were complex, differing depending upon the subgroup of the population. The prevalence of obesity among youths living in households headed by college graduates was lower than that among those living in households headed by less educated persons for each race-Hispanic origin group. The same was not true for those living in the highest income group. Moreover, differences by income and education of household head are widening among females.

Similar to results based on data from 2005 to 2008 (*4*), during 2011–2014 childhood obesity prevalence was lower among youths living in households in the highest income group. However, this was not the pattern seen in all subgroups. For example, obesity prevalence was lower in the highest income group compared with the other groups among non-Hispanic white females, but not among non-Hispanic black females, non-Hispanic white males, or non-Hispanic black males. Obesity prevalence decreased as head of household education increased in all subgroups examined. The prevalence of obesity was consistently lowest among children in households headed by college graduates, which differed from the pattern seen by income level. This difference in the relationship between obesity and income versus education has been observed in at least one other study (*8*). In addition, some relationships changed since 2005–2008. For example, there was a significant decreasing trend in obesity prevalence by income among non-Hispanic white males during 2005–2008 (*4*) but there were no differences during 2011–2014.

This report also presents differences in childhood obesity prevalence by income and education among non-Hispanic Asian youths in the United States. It has been suggested that the cut-off point that typically defines obesity might underestimate associated health risks among Asian persons (*9*).

The findings in this report are subject to at least one limitation. The sample size was small among some subgroups, such as non-Hispanic Asian females living in households with income above 350% of the FPL, where the prevalence of obesity is very low (1.3%) and the sample size is small (138). Additional years of data might provide more information about obesity prevalence by income, especially among non-Hispanic Asian youths.

Trends in childhood obesity prevalence by income and education level of head of household indicate that disparities have existed at least since NHANES III, 1988–1994 (*10*). These differences have widened since 1999–2002 among females but not among males, where differences in obesity prevalence by income and education of the head of household have remained relatively constant from 1999–2002 to 2011–2014.

These findings demonstrate that lower levels of income are not universally associated with childhood obesity. The association is complex and differs by sex, race, and Hispanic origin, and possibly over time. Differences by education are more consistent across subgroups than differences by income. More progress is needed to reduce disparities in childhood obesity prevalence, an important *Healthy People 2020* objective.

**Summary**

**What is already known about this topic?**

Studies have suggested that childhood obesity prevalence varies by income and education, although patterns might differ between adults and youths.

**What is added by this report?**

Analysis of data from the 2011–2014 National Health and Nutrition Examination Survey (NHANES) demonstrates that childhood obesity prevalence patterns among persons aged 2–19 years by household income are less consistent by race and Hispanic origin than are the patterns by level of education attained by the head of household. Moreover, the differences in childhood obesity prevalence by income and education of household head are widening among females while differences among males have remained relatively constant over time.

**What are the implications for public health practice?**

NHANES will continue to be an important source of data for monitoring disparities in childhood obesity. These data will help track the *Healthy People 2020* objective of reducing disparities and might inform obesity prevention programs at the federal, state, and local levels.
